# Activating enhancer binding protein 2 epsilon (AP-2ε)-deficient mice exhibit increased matrix metalloproteinase 13 expression and progressive osteoarthritis development

**DOI:** 10.1186/s13075-015-0648-8

**Published:** 2015-05-12

**Authors:** Stephan Niebler, Thomas Schubert, Ernst B Hunziker, Anja K Bosserhoff

**Affiliations:** Institute of Biochemistry (Emil-Fischer-Center), Friedrich Alexander University Erlangen-Nürnberg, Fahrstrasse17, 91054 Erlangen, Germany; Institute of Pathology, University Regensburg, Franz-Josef-Strauss-Allee 11, 93053 Regensburg, Germany; Institute of Pathology, Friedrich Alexander University Erlangen-Nürnberg, Krankenhausstrasse 8-10, 91054 Erlangen, Germany; Department of Orthopedic Surgery, University Hospital of Bern, Murtenstrasse 35, 3010 Bern, Switzerland

## Abstract

**Introduction:**

The transcription factor activating enhancer binding protein 2 epsilon (AP-2ε) was recently shown to be expressed during chondrogenesis as well as in articular chondrocytes of humans and mice. Furthermore, expression of AP-2ε was found to be upregulated in affected cartilage of patients with osteoarthritis (OA). Despite these findings, adult mice deficient for AP-2ε (*Tfap2e*^−/−^) do not exhibit an obviously abnormal cartilaginous phenotype. We therefore analyzed embryogenesis of *Tfap2e*^−/−^ mice to elucidate potential transient abnormalities that provide information on the influence of AP-2ε on skeletal development. In a second part, we aimed to define potential influences of AP-2ε on articular cartilage function and gene expression, as well as on OA progression, in adult mice.

**Methods:**

Murine embryonic development was accessed via *in situ* hybridization, measurement of skeletal parameters and micromass differentiation of mesenchymal cells. To reveal discrepancies in articular cartilage of adult wild-type (WT) and *Tfap2e*^−/−^ mice, light and electron microscopy, *in vitro* culture of cartilage explants, and quantification of gene expression via real-time PCR were performed. OA was induced via surgical destabilization of the medial meniscus in both genotypes, and disease progression was monitored on histological and molecular levels.

**Results:**

Only minor differences between WT and embryos deficient for AP-2ε were observed, suggesting that redundancy mechanisms effectively compensate for the loss of AP-2ε during skeletal development. Surprisingly, though, we found matrix metalloproteinase 13 (Mmp13), a major mediator of cartilage destruction, to be significantly upregulated in articular cartilage of adult *Tfap2e*^−/−^ mice. This finding was further confirmed by increased Mmp13 activity and extracellular matrix degradation in *Tfap2e*^−/−^ cartilage explants. OA progression was significantly enhanced in the *Tfap2e*^−/−^ mice, which provided evidence for *in vivo* relevance. This finding is most likely attributable to the increased basal Mmp13 expression level in *Tfap2e*^−/−^ articular chondrocytes that results in a significantly higher total Mmp13 expression rate during OA as compared with the WT.

**Conclusions:**

We reveal a novel role of AP-2ε in the regulation of gene expression in articular chondrocytes, as well as in OA development, through modulation of Mmp13 expression and activity.

## Introduction

Most components of the vertebrate skeleton are formed through a complex, multistep process termed *endochondral ossification* [1]. This process starts during early embryonic development, when mesenchymal cells condense at specific locations and prefigure future skeletal elements [2]. Subsequent differentiation of these cells results in chondrocytes that synthesize an abundance of extracellular matrix (ECM) proteins, including collagen type II and proteoglycans (for example, aggrecan). A premature skeletal template is thereby formed, entirely composed of cartilage [3]. After further differentiation steps, the cells become hypertrophic, attract blood vessels and ultimately undergo apoptosis, allowing osteoblasts to infiltrate the cartilaginous matrix to induce formation of trabecular bone [4, 5]. Endochondral ossification is completed in adults when most of the skeleton is replaced by bone tissue. However, in all diarthritic joints, a thin layer of permanent cartilage tissue remains intact throughout the organism’s lifetime. This “articular cartilage” acts as a shock absorber, minimizing peak pressures on the subchondral bone, and provides a smooth, low-friction gliding surface for efficient joint movement [6].

Chondrocytes are essential for physiological cartilage homeostasis. Under normal conditions, they maintain a constant, albeit low-level, equilibrium of matrix synthesis and degradation of ECM molecules. The latter is mediated by proteolytic enzymes such as matrix metalloproteinases (MMPs) and aggrecanases [7]. According to the prevailing hypothesis, disruption of this tightly controlled balance is one of the initial steps in osteoarthritis (OA) development [7–9]. OA is the most common joint disorder in Western populations. It is mainly described as an intrinsic, chronic disease of the articular cartilage (primary OA) [10, 11]. It is characterized by progressive degradation of the tissue, accompanied by biochemical and metabolic changes within the chondrocytes [12]. The exact molecular mechanisms that are responsible for OA onset are still poorly understood.

The initiation of OA often occurs on cartilage surface areas receiving the greatest mechanical forces. Eventually, chondrocytes begin to upregulate the production of ECM-degrading enzymes that mediate collagen and proteoglycan depletion. Here, members of the MMP family are attributed the most important role. MMPs comprise a group of zinc- and calcium-dependent endopeptidases that function as collagenases and aggrecanases. Of those, MMP13 (collagenase 3) is characterized as the central catabolic mediator that is overexpressed in nearly all cases of human OA, whereas other MMP variants only irregularly show enhanced expression [13–15]. In addition, several studies have demonstrated constitutive, albeit low-grade, expression of MMP13 in healthy human, rat and pig articular cartilage participating in physiologic ECM turnover [8, 14, 16, 17].

After a certain period, OA-induced damage of the articular cartilage becomes histologically obvious. Beginning with fissures and clefts in the cartilage surface, the defects quickly expand to the full depth of the tissue. Following staining with cationic dyes, local proteoglycan depletion is observed in the cartilage matrix bordering lesions [18]. Last, during end-stage OA, the hyaline cartilage is extensively eroded, and eburnated subchondral bone is formed. In addition, osteophyte formation at the joint margins can be observed [18, 19].

Recently, we revealed mRNA expression of the transcription factor activating enhancer binding protein 2 epsilon (*AP-2ε*) in chondrocytes and during chondrogenic differentiation of human mesenchymal stem cells. Additionally, we detected AP-2ε *in vivo* in murine hypertrophic chondrocytes during embryogenesis and in human articular cartilage via immunohistochemistry [20, 21]. AP-2ε is a member of the AP-2 transcription factor family, which consists of five isoforms (AP-2α to AP-2ε) and influences a vast number of physiological and pathogenic processes [22–24]. AP-2ε was also found to be upregulated in articular cartilage of OA patients [21]. Furthermore, *in vitro*, AP-2ε induced the expression of chemokine (C-X-C) motif ligand 1 (*CXCL1*) [25] and inhibited *COL2A1* (encoding collagen type II) in human chondrocytic cells [26]. Despite these findings, adult mice deficient for AP-2ε (*Tfap2e*^−/−^) do not show an obvious cartilage phenotype under normal physiological conditions.

Therefore, in the present study, we analyzed possible roles of AP-2ε in embryonic skeletal development, as well as in articular cartilage function after the surgical induction of OA, using the *Tfap2e*^−/−^ mouse. The obtained results will help to enhance understanding of the functional role of AP-2ε in cartilage homeostasis in health and disease, as well as knowledge of OA progression in general.

## Methods

### Transgenic mice, tissue preparation and animal studies

*Tfap2e*^−/−^ mice were generated and kindly provided by Markus Moser (Max Planck Institute for Biochemistry, Munich, Germany). In those mice, a neomycin DNA construct containing a stop codon was inserted into the second exon of the *Tfap2e* gene between the ApaII and NotI restriction sites (unpublished observations). This modification leads to a premature termination of the translation process of subsequent AP-2ε mRNA transcripts, resulting in a complete absence of functional AP-2ε protein in animals homozygous for this genetic mutation.

*Tfap2e*^−/−^ and the corresponding wild-type (WT) mice were bred at 26 °C and 70 % relative humidity under a 12-hour light/12-hour dark cycle at the University Hospital of Regensburg, Germany. The mice were fed a breeding and maintenance diet (Altromin Spezialfutter GmbH, Lage, Germany) and given water *ad libitum*. The mice were randomly housed in polypropylene cages with sawdust bedding, and the cages were sanitized twice weekly. Animal care and all experimental procedures were carried out in accordance with guidelines under the German law governing animal care. The OA surgery was approved by the Ethics Committee for Animal Research of the Bavarian government. For all other experiments in which murine tissue was used, it was sufficient to obtain supervision from the local animal welfare officer (Dr Thilo Spruss, University Hospital Regensburg) according to the German Animal Welfare Act 2006 (article 4, using mice for scientific purposes (including tissue, embryo and cell extraction) if no additional experimental procedures are carried out with the animals). Therefore, except of the OA model, no further notification or approval by the Ethics Committee for Animal Research of the Bavarian government was necessary for the mouse studies.

All adult mice were killed after anesthetization by isoflurane (2-chloro-2-(difluoromethoxy)-1,1,1-trifluoroethane) inhalation via cervical dislocation. Embryos were killed by decapitation. To generate histological sections, tissue and embryos were fixed for at least 3 days in phosphate-buffered saline (PBS) containing 4 % paraformaldehyde, decalcified in 20 % ethylenediaminetetraacetic acid (Sigma-Aldrich Chemie GmbH, Munich, Germany) if necessary and embedded in paraffin. Subsequently, 5-μm serial sections were cut. For genotyping, tail biopsies were used.

To obtain mouse embryos of a certain age, adult pubescent mice were coupled overnight, and the weight of the females was documented. At the desired time point, the pregnant mice were killed after successful pregnancy was determined on the basis of a clear weight increase. Embryos were harvested by carefully opening the abdominal wall and uterus.

To obtain adult articular cartilage of murine knee joints, the joints were dissected and the cartilage layer of the femoral condyles and the tibial plateau were carefully separated from the underlying bone using sharp scissors and a scalpel. Total RNA was isolated after pulverization of the cartilage using liquid nitrogen and a mortar.

OA in 6- to 8-week-old WT and *Tfap2e*^−/−^ mice was induced by detaching the medial meniscus (DMM) from the tibial plateau as described previously [27]. Briefly, with the mice under general anesthesia using a 0.9 % sodium chloride solution containing 0.75 % ketamine and 0.16 % xylazine (10-μl intraperitoneal injection per gram of body weight), the hind limbs were prepared for aseptic surgery. The right knee capsule was exposed following a medial incision without transection of the patellar ligament. Next, the medial meniscus was detached from the tibial plateau using a fine cannula and a surgical microscope. As a result, the meniscus translocated freely into the joint space. Afterward, the skin was closed with a metal clamp. During the procedure, close attention was paid not to injure the articular cartilage surface. In the left knee joint, the capsule was opened without further treatment and served as a control (sham). The animals were killed 10 or 17 days after surgery, and the knee joints were prepared for histological sectioning or RNA isolation from articular cartilage. The progress of OA was scored by stage and grade according to the scoring system proposed by Pritzker *et al.* [18] using sections stained with Safranin O/Fast Green and hematoxylin and eosin, respectively. In a preliminary test, no significant differences in OA development between male and female mice could be observed. Thus, both sexes were used in equal numbers.

### Preparation of genomic DNA and genotyping

Whole genomic DNA extracts were isolated from tail biopsies as well as from hip cartilage explants using the QIAamp DNA Mini Kit (Qiagen, Hilden, Germany) as described by the manufacturer. The DNA concentration and purity were measured using a NanoDrop device (Peqlab Biotechnologie GmbH, Erlangen, Germany). *Tfap2e* genotyping was performed using a PTC-200 thermocycler (MJ Research, Waltham, MA, USA). The following PCR program was used: 5 minutes at 95 °C (initial denaturation); 30 seconds at 95 °C (denaturation), 30 seconds at *x* °C (annealing) (Table 1) and 30 seconds at 72 °C (elongation), repeated 10 times, beginning with an annealing temperature of 63 °C and 1 °C reduction per each subsequent cycle in a total volume of 50 μl containing 2 μl of genomic DNA template, 1.5 μl of a mixture of three specific primers (20 μM; Sigma-Aldrich Chemie GmbH) (Table 1), which bound to the *Tfap2e* genomic locus and the neomycin insert, as well as 25 μl of FailSafe PCR 2× PreMix D (Epicentre, Madison, WI, USA) and 0.5 μl of Taq DNA polymerase (Roche Diagnostics GmbH, Mannheim, Germany). The initial PCR was followed by 30 additional cycles of 30 seconds at 95 °C, 30 seconds at 53 °C and 30 seconds at 72 °C. The PCR products were evaluated by performing gel electrophoresis in a 1.5 % agarose gel. Running the genomic DNA of homozygous *Tfap2e*^−/−^ mice on the gel resulted in a 300-bp band; DNA from homozygous WT mice resulted in a 100-bp band; and DNA of heterozygous animals produced both bands.

**Table 1:** Primers used for genotyping of wild-type and Tfap2e^−/−^ mice^a^

Primer	Sequence (5′-3′)
mAP-2ε gen anti2	CTACGTCGCCCTGGACTTCGAGC
mAP2ε gen sense2	TGGAATCCTGTGGCATCCATGAAAC
pGK polyA down	GGCTCTCCAGAACATCATCCCTGC

^a^Sequences were obtained from M Moser

### RNA isolation, reverse transcription and quantitative real-time PCR

Total RNA of murine tissues was isolated using a E.Z.N.A. MicroElute Total RNA Kit (Omega Bio-Tek, Norcross, GA, USA) as described by the manufacturer. The purity and concentration of the RNA were measured using a NanoDrop device (Peqlab Biotechnologie GmbH), and cDNA was generated by reverse transcription as described elsewhere [28]. Each reaction was performed in a total volume of 20 μl containing at least 150 ng of total RNA.

Quantitative RT-PCR (qRT-PCR) was carried out using the LightCycler 480 system from Roche Diagnostics GmbH. Volumes of 1 μl of cDNA template, 0.5 μl of forward and reverse primers (20 mM), 10 μl of SYBR Green Premix (Roche Diagnostics GmbH) and 8 μl of water were combined in a total volume of 20 μl. Primers were obtained from Sigma-Aldrich Chemie GmbH and are listed in Table 2. The following PCR program was used: 95 °C for 10 minutes (initial denaturation); 4.4 °C per second temperature transition rate up to 95 °C for 10 seconds, *x* °C for 10 seconds (annealing) and 72 °C for 20 seconds (elongation), and *y* °C acquisition mode single, repeated 45 times (amplification). The annealing (*x*) and acquisition (*y*) temperatures were optimized for each primer set. The PCR product was evaluated using melting curve analysis, and each sample was analyzed at least in duplicate. The expression level of the analyzed genes was normalized to the expression level of the housekeeping gene *Actb* (encoding β-actin).

**Table 2:** Murine primer pairs used for quantitative real-time PCR

Gene	Primer	Product	Sequence (5′-3′)
*Actb*	mβ-Act_885for	348 bp	TGGAATCCTGTGGCATCCATGAAAC
	mβ-Act_1233rev		TAAAACGCAGCTCAGTAACAGTCCG
*Acan*	mAggrecan_1922for	206 bp	CAGTTCACCTTCCAGGAAG
	mAggrecan_2128rev		GTAGAGGTAGACCGTTCTCACG
*Col2a1*	mColl2_2657for	261 bp	CTACTGGAGTGACTGGTCCTAAGG
	mColl2_2918rev		GGACCATCATCTCCAGGTTCTCC
*Col10a1*	mCol10_38for	287 bp	CTGCCCCACGCATCTCCCAG
	mCol10_325rev		GCTTGCCTGGCGGTCCTGAG
*Mmp1a/Mmp1b*	mMMP1_17for	175 bp	CTGTTGCTTCTCTGGGCTGC
	mMMP1_192rev		CTGCATTTGCCTCAGCTTTTC
*Mmp3*	mMMP3_362for	104 bp	GTTCCTGATGTTGGTGGCTTCAG
	mMMP3_466rev		CTGTCTTGGCAAATCCGGTGTA
*Mmp13*	mMMP13_899for	80 bp	CCCAGCCCTATCCCTTGATGCCA
	mMMP13_979rev		TGCAGGCGCCAGAAGAATCTGT
*Tfap2a*	mAP-2α_1170for	69 bp	GCGGCCCAATCCTATCCT
	mAP-2α_1238rev		CCATGGGAGATGAGGTTGAAG
*Tfap2b*	mAP-2β_1355for	67 bp	AAAGCTGTCTCACGCACTTCAGT
	mAP-2β_1421rev		AGCGCAGCGCAAATGG
*Tfap2c*	mAP-2γ_1115for	537 bp	ACCTAGCACGGGACTTCGCCT
	mAP-2γ_1651rev		GGGCGGGCGGGTTGTAACTG
*Tfap2e*	mAP-2ε_388for	154 bp	GCCGACCCTGGGGAGCTACAC
	mAP-2ε_542rev		CACCTCCGGCGCCGCTTAAA
*Timp1*	mTIMP1_147for	270 bp	AGACACACCAGAGCAGATACC
	mTIMP1_417rev		CCGGATATCTGCGGCATTTC
*Timp2*	mTIMP2_442for	280 bp	GCAGACGTAGTGATCAGAGCC
	mTIMP2_722rev		TCCCAGGGCACAATGAAGTC
*Timp3*	mTIMP3_255for	315 bp	GACCCTTGGCCACTTAGTCC
	mTIMP3_570rev		CGGATCACGATGTCGGAGTTG

### *In situ* hybridization

For *in situ* hybridization of paraffin-embedded sections, digoxigenin-labeled antisense RNA probes that were specific for mouse *Col2a1* (collagen, type II, alpha 1) and *Col10a1* (collagen, type X, alpha 1) mRNA transcripts were generated using reverse transcription. The corresponding vectors, which were kindly provided by Klaus von der Mark (University of Erlangen-Nürnberg, Erlangen, Germany), were linearized using EcoRI (for *Col2a1*) and BamHI (for *Col10a1*) restriction enzymes, and the antisense RNA probes were subsequently reverse-transcribed using T3 RNA polymerase (for *Col2a1*) or T7 RNA polymerase (for *Col10a1*) with digoxigenin-labeled deoxynucleoside triphosphates (Roche Diagnostics GmbH). Serial sections of the hind limbs from WT and *Tfap2e*^−/−^ embryos were analyzed using *in situ* hybridization as described elsewhere [29]. RNA probes bound to the tissue were detected using an anti-digoxigenin alkaline phosphatase antibody and BM purple (both purchased from Roche Diagnostics GmbH) according to the manufacturer’s instructions. The sections were mounted with coverslips in Kaiser’s glycerol gelatin (Merck, Darmstadt, Germany).

### *In vitro* micromass cultivation of embryonic limb bud cells

Twelve to twenty-four limb buds that were derived from littermates of pregnant homozygous mice were pooled and dissolved in Dulbecco’s modified Eagle’s medium (DMEM)/Ham’s F-12 (1:1) (PAA, Pasching, Austria) containing dispase (1 U/ml) (Life Technologies, Carlsbad, CA, USA), 10 % fetal calf serum (FCS; PAN Biotech GmbH, Aidenbach, Germany), penicillin (100 U/ml; Sigma-Aldrich Chemie GmbH) and streptomycin (10 μg/ml; Sigma-Aldrich Chemie GmbH) at 37 °C for 30 minutes. Single cells were collected by passaging through a 40-μm filter and centrifugation at 280 × *g* for 4 minutes. Afterward, the cells were resuspended in DMEM/Ham’s F-12 (1:1) containing 10 % FCS and penicillin-streptomycin at a concentration of 20,000 cells/μl. Ten microliters of this suspension were applied to each well of a 24-well plate, which was then incubated for 3 hours at 37 °C and 5 % CO_2_ for cell attachment. Subsequently, each well was carefully flooded with 800 μl of DMEM/Ham’s F-12 (1:1) supplemented with 10 % FCS, penicillin-streptomycin, l-ascorbic acid 2-phosphate (100 mM; Sigma-Aldrich Chemie GmbH) and β-glycerophosphate (100 mM; Sigma-Aldrich Chemie GmbH). The cultures were incubated for up to 4 days, and the media were changed daily. Directly after establishment (d0) and on each following day, the cells of two wells were harvested for RNA isolation. Additionally, on each day, a culture was stained with 1 % Alcian Blue (Sigma-Aldrich Chemie GmbH) in 0.1 M HCl for 2 hours after fixation with a solution containing 30 % EtOH, 0.37 % formaldehyde and 3.9 % acetic acid. After removal of excessive Alcian Blue with PBS and 70 % EtOH, the characteristic nodules that resembled chondrocytic differentiation became visible.

### *In vitro* cultivation of articular cartilage explants

To obtain cartilage explants from the hip joints, the pan-shaped articular cartilage that coated the femoral head was carefully pulled off the underlying bone using a fine forceps. From each mouse, two explants were isolated, and their weights were determined. The explants were cultured in 24-well plates at 37 °C and 5 % CO_2_ in 500 μl of DMEM/high glucose (PAA) containing 10 % FCS (PAN Biotech GmbH), penicillin (100 U/ml; Sigma-Aldrich Chemie GmbH), streptomycin (10 μg/ml; Sigma-Aldrich Chemie GmbH) and amphotericin B (0.5 μg/ml; Sigma-Aldrich Chemie GmbH). After 2 days, the medium was exchanged to serum-free medium (supplemented as before), and a specific Mmp13 inhibitor (pyrimidine-4,6-dicarboxylic acid, *bis*-(4-fluoro-3-methyl-benzylamide); Merck) was added to a number of the explants at a concentration of 100 nM (one explant with and one without inhibitor per mouse). After 4 days at 37 °C and 5 % CO_2_, the supernatants were collected, and Mmp13 activity and glycosaminoglycan (GAG) concentration were measured. Additionally, genomic DNA of the remaining explants was isolated, and their concentrations were measured to indicate the number of chondrocytes in the cartilage fragments. This value and the weight of the explants were used to normalize the absolute GAG concentration and Mmp13 activity in the supernatants.

### Measurement of matrix metalloproteinase 13 activity and glycosaminoglycan concentration in media

Analysis of Mmp13 activity in media was performed using the SensoLyte 520 MMP-13 Assay Kit *Fluorimetric* (AnaSpec, Fremont, CA, USA) according to the manufacturer’s instructions. The fluorescence intensity, which directly correlated with Mmp13 activity, was determined using a FLUOstar Omega fluorescence reader (BMG LABTECH, Ortenberg, Germany) at excitation/emission wavelengths of 485/520 nm.

For the detection of GAG, the WIESLAB sGAG quantitative kit was used (Euro-Diagnostica, Malmö, Sweden) as suggested by the manufacturer. The color intensity, which represented the GAG concentration, was measured using a plate reader (MWG Biotech, Ebersberg, Germany) at 650 nm.

### Immunohistochemical staining of collagen cleavage neoepitopes

Decalcified, paraffin-embedded sections of murine knee joints were deparaffinized, rehydrated and incubated with chondroitinase ABC (Sigma-Aldrich Chemie GmbH) at 0.025 U/100 μl in 0.1 M Tris-HCl (pH 8.0) containing 60 mM sodium acetate and 0.02 % bovine serum albumin for 90 minutes at 37 °C to remove GAG. Endogenous peroxidase activity was quenched by treating the sections with 1 % H_2_O_2_ for 3 minutes. For the detection of collagenase-induced generation of collagen type 2 neoepitopes in the cartilage matrix, a rabbit antiserum raised against the C1,2C (COL2 3/4C_short_) neoepitope was used (IBEX Technologies, Montreal, QC, Canada). Sections were incubated with the primary antibody (1:100) for 30 minutes at room temperature. After extensive rinsing, the secondary antibody Histofine Simple Stain MAX PO anti-rabbit (Nichirei Biosciences, Tokyo, Japan) was incubated for 30 minutes at room temperature and subsequently visualized using diaminobenzidine chromogen (Dako, Hamburg, Germany). Finally, the tissue was counterstained with hematoxylin. The specificity of the staining was controlled with sections of human OA cartilage as positive controls and sections without primary antibody as negative controls.

### Light and transmission electron microscopy

Light microscopy was performed using a Zeiss Axiovert200 microscope, which was equipped with an AxioCam MRc camera (Carl Zeiss, Jena, Germany). AxioVs40 V 4.5.0.0 software (Carl Zeiss) was applied to quantify the zones of gene expression after *in situ* hybridization, as well as to measure skeletal parameters.

Transmission electron microscopy was conducted in collaboration with Josef Schroeder and Heiko Siegmund (University Hospital of Regensburg, Institute for Pathology, Regensburg, Germany). For this purpose, the femora and tibiae of 6- to 7-week-old WT and *Tfap2e*^−/−^ mice were isolated and chemically fixed for 16 hours in Karnovsky’s reagent (2.5 % glutaraldehyde/1 % paraformaldehyde). Articular cartilage of the knee joint was processed as described earlier [30, 31]. The transmission electron micrographs were qualitatively evaluated by Ernst B Hunziker. For this purpose, pictures derived from equal articular cartilage zones were compared.

### Statistical analysis

The results are expressed as the mean ± standard error of the mean or as box-and-whisker plot with minimum and maximum. Comparison between groups was made using Student’s paired or unpaired *t*-test, and a *P*-value <0.05 was considered statistically significant. All calculations were performed using GraphPad Prism software (GraphPad Software, La Jolla, CA, USA).

## Results

### *Tfap2e*^−/−^ mice exhibit only minor abnormalities in cartilage and skeletal development during embryogenesis

*AP-2ε* expression was recently detected in human articular cartilage and in hypertrophic chondrocytes of embryonic day 14.5 (E14.5) WT mouse embryos [20, 21]. However, adult *Tfap2e*^−/−^ mice showed no obvious phenotype, and the exact role of the transcription factor in cartilage development and homeostasis was still unknown. Therefore, in the first part of this study, we examined the embryonic development of the *Tfap2e*^−/−^ mouse for potential transient abnormalities that might be compensated later and could provide information about the influence of the transcription factor on chondrogenesis.

**Fig. 1:**
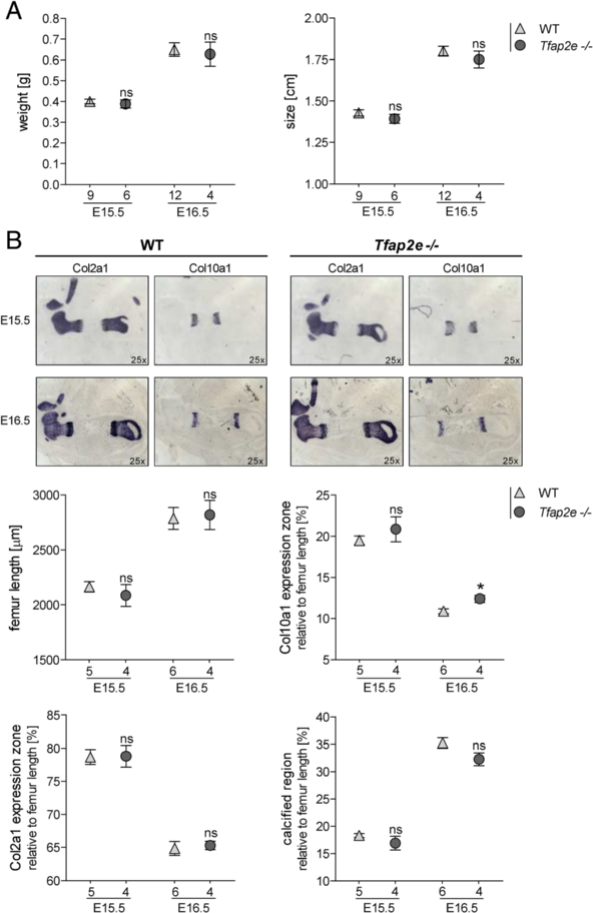
Comparison of the embryonic skeletal development in embryonic days 15.5 and 16.5 wild-type and *Tfap2e* ^−/−^ mice. **a** The total weight and size of 15.5- and 16.5-day-old (E15.5 and E16.5, respectively) wild-type (WT) and *Tfap2e* ^−/−^ embryos derived from three independent heterozygote litters per time point were approximately identical. **b** *In situ* hybridization against collagen, type II, alpha 1 (*Col10a1*) and collagen, type X, alpha 1 (*Col10a1*) mRNA on the hind limbs of the embryos and subsequent measurement of the total femur length, the *Col2a1* and *Col10a1* expression zones and the bone region was performed. The latter three were normalized to total femur length; the distal and proximal expression zones of *Col2a1* and *Col10a1* were combined before normalization. A slight but significant increase in the *Col10a1* expression zone could be observed in the *Tfap2e* ^−/−^ fetuses at E16.5. Otherwise, no evidence for abnormal development in the *Tfap2e* ^−/−^ mice could be determined. The data are given as the means ± standard error of the mean. ns, Not significant; *Tfap2e* ^−/−^, Deficient for activating enhancer binding protein 2, epsilon. *P < 0.05. Numbers indicate individual embryos used for each group

We initially concentrated on E15.5 and E16.5, as these stages are particularly suited to assessment of chondrocyte differentiation and endochondral ossification in the murine limbs. For this purpose, heterozygous adult mice were coupled overnight, and homozygous littermates derived from the same female mouse were compared. This ensured that all *Tfap2e*^−/−^ and WT embryos were of identical age and avoided unequal development due to divergent mother animals. No significant differences in total weight or size between the two genotypes could be observed (Fig. 1a). Additionally, the hind limbs of the embryos were embedded in paraffin, and sections were stained for *Col2a1* and *Col10a1* mRNA expression via *in situ* hybridization to more closely analyze limb development (Fig. 1b). The expression zones of the two genes were measured and normalized to the femur length. As depicted in Fig. 1b, no significant differences between the femora of WT and *Tfap2e*^−/−^ specimens in total length, *Col2a1* expression zone or calcified region (bone) could be observed. However, the *Col10a1* expression zone at E16.5 in *Tfap2e*^−/−^ embryos was slightly but significantly enhanced.

**Fig. 2:**
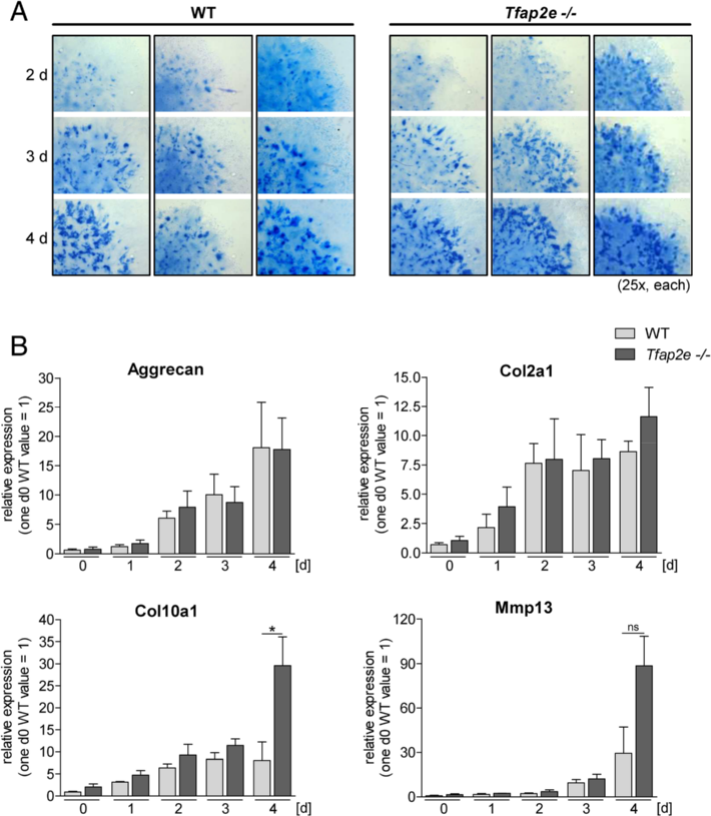
Micromass *in vitro* differentiation of mesenchymal limb bud cells. Micromass cultures of mesenchymal cells isolated from the limb buds of embryonic day 11.5 (E11.5) wild-type (WT) and *Tfap2e* ^−/−^ embryos were maintained for 4 days *in vitro*. **a** To compare the differentiation behavior of WT and *Tfap2e* ^−/−^ cells, each day, a culture was treated with Alcian Blue solution to stain for secreted glycosaminoglycans. The nodules that are characteristic for this assay became visible at the second day of the culture period. Their number, size and color intensity increased in both genotypes by approximately the same amount. **b** Directly after initiation of the cultures (d0) and on each following day, mRNA expression of the cartilage differentiation markers aggrecan, collagen, type II, alpha 1 (*Col10a1*) and collagen, type X, alpha 1 (*Col10a1*) and matrix metalloproteinase (*Mmp13*) was analyzed by quantitative RT-PCR. A definite increase in the expression level of all four genes during the differentiation process was detectable in cells of both genotypes. The expression of *Col10a1* was enhanced, and expression of *Mmp13* tended to be enhanced, at day 4 in *Tfap2e* ^−/−^ cells compared with WT cells. The data are given as the means ± standard error of the mean. ns, Not significant; *Tfap2e* ^−/−^, Deficient for activating enhancer binding protein 2, epsilon. *P < 0.05. The assay was carried out four times with cells of four individual WT litters and three times with cells of three individual *Tfap2e* ^−/−^ litters

To define the impact of AP-2ε on earlier steps of embryonic cartilage development, we used mesenchymal cells isolated from limb buds of E11.5 WT and *Tfap2e*^−/−^ mouse embryos in high-density micromass cultures. The production and accumulation of sulfated GAG was determined by staining the cultures with Alcian Blue solution. Formation of the typical nodules could be observed 2 days after initiation of the cultures (Fig. 2a). Subsequently, the number and color intensity of the nodules increased, but no definite discrepancy between the two genotypes was observed. Additionally, the expression patterns of prominent differentiation marker genes in cartilage (aggrecan, *Col2a1*, *Col10a1* and *Mmp13*) were compared by qRT-PCR (Fig. 2b). No differences between *Tfap2e*^−/−^ and WT cells could be observed for aggrecan or *Col2a1*; however, expression of *Mmp13* and *Col10a1* was increased in *Tfap2e*^−/−^ cultures in late stages of differentiation.

In sum, these data imply that AP-2ε most likely does not play a major role during embryonic cartilage development in mice. Alternatively, other factors may effectively compensate for the loss of AP-2ε in the knockout animals during embryogenesis. Nevertheless, the results suggest that expression of the genes for *Mmp13* and *Col10a1* could be affected by the loss of *AP-2ε* in differentiated chondrocytes. To further support this hypothesis, we expanded our investigation of the *Tfap2e*^−/−^ mouse to postnatal articular cartilage tissue in the second part of this study.

### Expression and activity of the proteinase matrix metalloproteinase 13 is upregulated in articular cartilage of *Tfap2e*^−/−^ mice

**Fig. 3:**
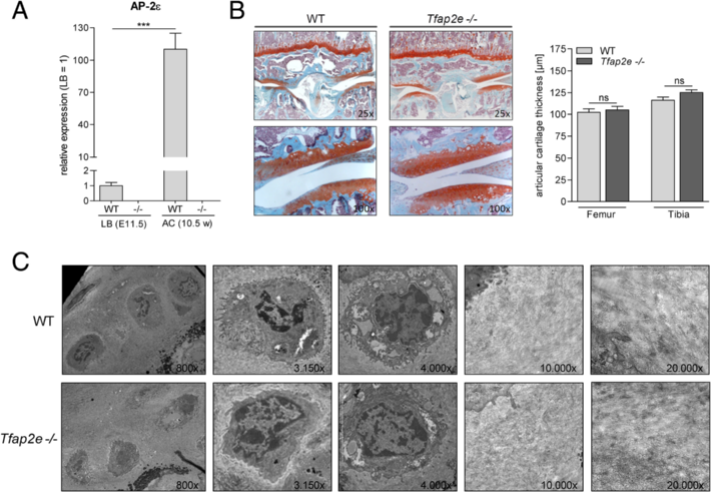
Activating enhancer binding protein 2, epsilon, expression in articular cartilage and analysis of the cartilage layer in wild-type and *Tfap2e* ^−/−^ mice. **a** Activating enhancer binding protein 2, epsilon (AP-2ε) mRNA expression was measured in mesenchymal limb bud cells (LB) of embryonic day 11.5 (E11.5) embryos and in articular chondrocytes (AC) from the knee joints of 10.5-week-old (10.5 w) mice via quantitative RT-PCR. In the wild-type (WT) samples, a strong induction in the expression rate of the transcription factor was observed. As expected, when we used genotype-specific primers, normal AP-2ε mRNA without the neomycin insert could not be detected in corresponding *Tfap2e* ^−/−^ samples, serving as a negative control. **b** Histological sections through the knee joints of 10.5-week-old WT and *Tfap2e* ^−/−^ mice after staining with Safranin O/Fast Green (left). Morphologically, no obvious abnormalities between the two genotypes could be detected at this age. Furthermore, the thickness of the articular cartilage layer in the tibia and femur was similar in each genotype (right). **c** Exemplary pictures from the ultrastructural analysis of the articular cartilage layer in the knee joints of adult WT and *Tfap2e* ^−/−^ mice depicting chondrocytes (left) and the extracellular matrix (right). No obvious differences could be determined in a qualitative evaluation. The data are given as the means ± standard error of the mean. ns, Not significant; *Tfap2e* ^−/−^, Deficient for activating enhancer binding protein 2, epsilon. ***P < 0.001. In (A), cells of four individual litters were used for LB and nine individual animals for AC. In (B), 14 WT and 15 *Tfap2e* ^−/−^ mice were compared. In (c), two mice were used for each genotype. Micrographs shown are derived from the middle and deep zones of the cartilage layer and were adjusted to equal magnification

**Fig. 4:**
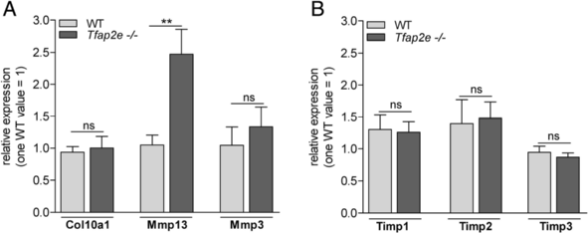
Gene expression analysis in articular chondrocytes of wild-type and *Tfap2e* ^−/−^ mice. **a** The expression of collagen, type X, alpha 1 (*Col10a1*), matrix metalloproteinase 13 (*Mmp13*) and *Mmp3* was analyzed in the articular cartilage layer of knee joints of wild-type (WT) and *Tfap2e* ^−/−^ mice (10.5 w) by quantitative RT-PCR. *Mmp13*, but not *Col10a1* or *Mmp3*, was significantly overexpressed in *Tfap2e* ^−/−^ mice. **b** mRNA expression of tissue inhibitor of metalloproteinase 1 (Timp1), Timp2 and Timp3 was analyzed in the articular cartilage samples, but no differences in the expression rates of the three molecules could be detected when we compared WT and *Tfap2e* ^−/−^ animals. The data are given as the means ± standard error of the mean. ns, Not significant; *Tfap2e* ^−/−^, Deficient for activating enhancer binding protein 2, epsilon. **P < 0.01. Nine WT mice and eight *Tfap2e* ^−/−^ mice were used for this experiment

Measuring the *AP-2ε* mRNA levels in articular chondrocytes of the knee joints of adult WT mice revealed a strong induction in expression compared with the mesenchymal limb bud cells used for micromass differentiation (Fig. 3a). This strongly hinted at a role of AP-2ε in the homeostasis of articular cartilage and/or the regulation of gene expression in this tissue type. However, initially, no evident abnormalities in the articular cartilage layer of 10.5-week-old *Tfap2e*^−/−^ mice could be determined at the histological or ultrastructural level based on a qualitative analysis (Fig. 3b and c). To analyze whether abnormal expression of another AP-2 isoform might effectively compensate for the loss of AP-2ε in articular cartilage of the knockout mice, expression of *AP-2α, AP-2β and AP-2γ* was determined, but no differences from WT animals could be measured (data not shown). Next, we analyzed *Mmp13* and *Col10a1* expression, as these two genes showed differential expression when we compared highly differentiated mesenchymal cells of WT and *Tfap2e*^−/−^ mice (see Fig. 2b). Interestingly, in articular chondrocytes deficient for AP-2ε, expression of *Mmp13* was significantly enhanced, whereas this was not the case for *Col10a1* (Fig. 4a).

To evaluate the specificity of the detected Mmp13 dysregulation, expression of *Mmp1 and Mmp3*, two other metalloproteinases that are known to play critical roles in cartilage development and in human OA, was also examined [7, 15]. Two highly conserved genes for the interstitial collagenase Mmp1, *Mmp1a* and *Mmp1b*, exist in mice, but the mRNA of neither was detectable in articular chondrocytes of WT and *Tfap2e*^−/−^ mice (data not shown). *Mmp3* was expressed, but no differences between the two genotypes could be determined (Fig. 4a). In addition, no differential expression of the genes for tissue inhibitors of metalloproteinase 1, 2 and 3 (*Timp1*, *Timp2* and *Timp3*, respectively) was determined (Fig. 4b). Taken together, this suggested that primarily *Mmp13* is dysregulated in articular chondrocytes of *Tfap2e*^−/−^ mice.

**Fig. 5:**
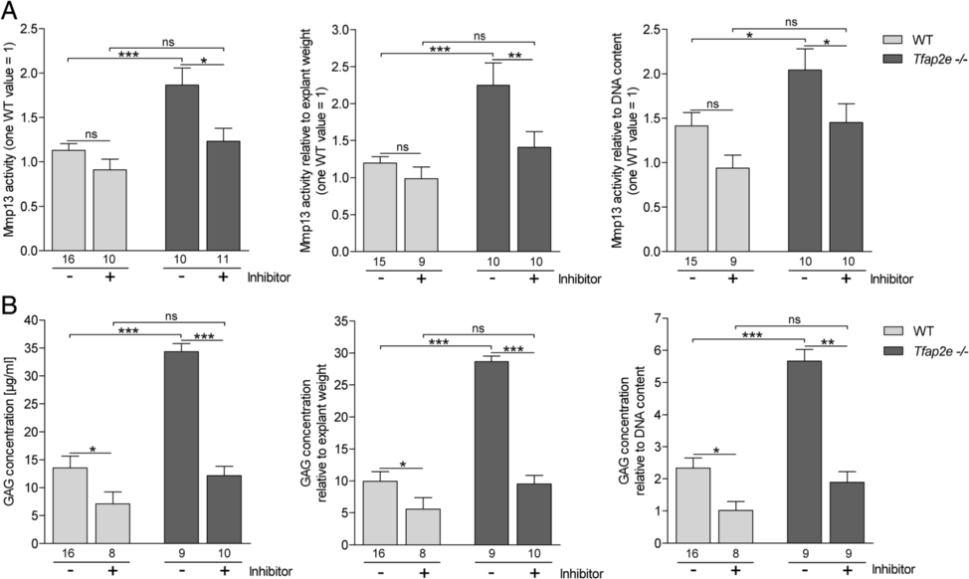
Enhanced matrix metalloproteinase 13 activity and matrix degradation in articular cartilage explants deficient for activating enhancer binding protein 2, epsilon. Articular cartilage of the femoral head of adult wild-type (WT) and *Tfap2e* ^−/−^ mice (two per animal) was isolated and subjected to *in vitro* cultivation. Two days after establishment, a specific matrix metalloproteinase (Mmp13) inhibitor was added to a subset of the explants. Another 3 days later, the supernatants were collected, and Mmp13 activity **(a)** and glycosaminoglycan (GAG) concentrations **(b)** were determined. Both were significantly enhanced in media from the *Tfap2e* ^−/−^ explants compared with the WT explants (left). Treatment with the Mmp13 inhibitor resulted in a significant reduction in Mmp13 activity and GAG release in the *Tfap2e* ^−/−^ explants. This was also detectable in the supernatants of WT explants, albeit at a lower level. In addition, the absolute values for both parameters were normalized to the total wet weight (middle), as well as to the DNA content (right), of the corresponding explants. Here, similar results were discovered. The data are given as the means ± standard error of the mean. ns, Not significant; *Tfap2e* ^−/−^, Deficient for activating enhancer binding protein 2, epsilon. *P < 0.05; **P < 0.01; ***P < 0.001. Numbers indicate individual explants used for each group

To provide evidence for enhanced catabolic activity in articular cartilage tissue of *Tfap2e*^−/−^ mice due to the observed overexpression of Mmp13, we performed *in vitro* culture of femoral head cartilage explants. After cultivation, Mmp13 activity in the supernatants was measured using a fluorescence-based assay. We detected significantly higher activity of the proteinase in medium derived from explants deficient for AP-2ε relative to WT explants (Fig. 5a, left). Furthermore, addition of a specific Mmp13 inhibitor resulted in a slight reduction of Mmp13 activity in the WT and a significant reduction nearly to the WT level in the *Tfap2e*^−/−^ explants.

Additionally, the GAG content in the supernatants, which is an indicator of proteoglycan and ECM degradation, was determined. Interestingly, the absolute GAG concentration was also significantly higher in the supernatants of the *Tfap2e*^−/−^ cartilage explants compared with WT cartilage explants (Fig. 5b, left). Furthermore, the Mmp13 inhibitor significantly reduced GAG release compared with the untreated explants. Similar results were obtained after normalization of the Mmp13 activity and the GAG concentration to the weight of the explants before cultivation, as well as to their DNA content, representing the number of cells in the cartilage fragments (Fig. 5a and b, middle and right). In sum, these *in vitro* data strongly support that expression and activity of Mmp13 is upregulated in articular chondrocytes of *Tfap2e*^−/−^ mice, resulting in enhanced matrix degradation.

### Enhanced osteoarthritis development in *Tfap2e*^−/−^ mice

To confirm this hypothesis *in vivo*, WT and *Tfap2e*^−/−^ mice were subjected to a model of OA. As we were unable to observe any morphological or ultrastructural abnormalities in the articular cartilage layer of 10.5-week-old *Tfap2e*^−/−^ mice under physiological conditions (see Fig. 3), we speculated that potential abnormalities in the AP-2ε-knockout mice become apparent under these conditions of stress. It is also known, on the basis of studying other mouse models deficient for cartilage-specific genes, that phenotypic variations sometimes become obvious only in pathological situations [27, 32–34].

**Fig. 6:**
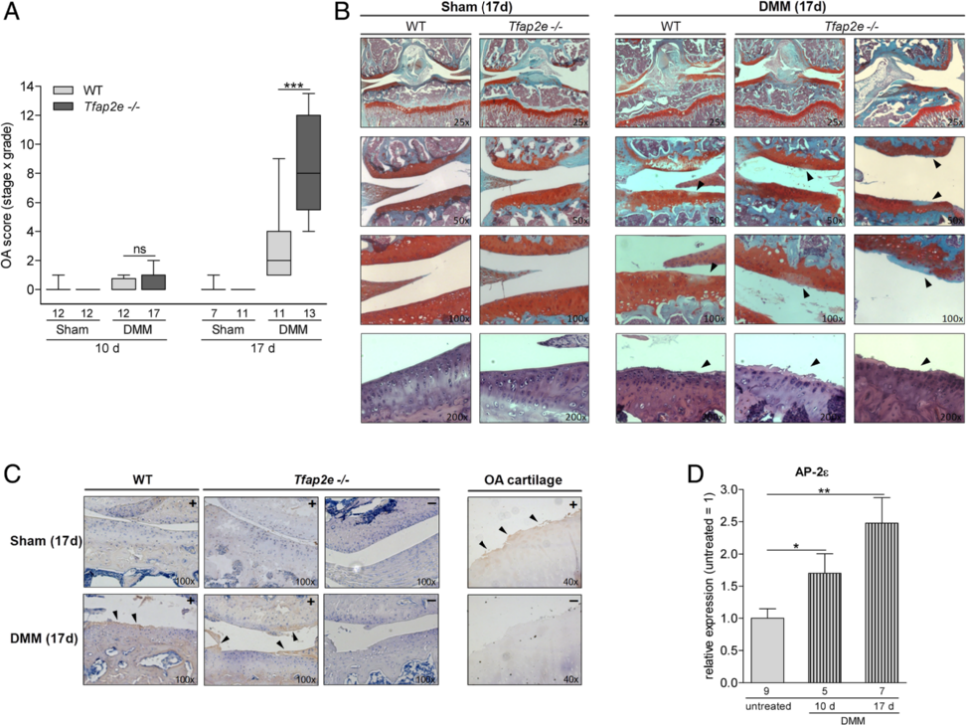
Enhanced osteoarthritis progression in *Tfap2e* ^−/−^ mice. **a** In 6- to 8-week-old wild-type (WT) and *Tfap2e* ^−/−^ mice, osteoarthritis (OA) was induced in the right knee joint by detaching the medial meniscus (DMM). In the corresponding left knee joint, a control surgery was performed (Sham). Ten days after surgery, all mice showed only minimal signs of OA. Seventeen days after disease onset, WT mice exhibited low to moderate OA scores, whereas OA severity in *Tfap2e* ^−/−^ mice was significantly enhanced at this time point. **b** Visible damage to the articular cartilage tissue at day 17 after DMM surgery included wear, lesions and inhomogeneous staining (right, arrowheads). Cartilage of the sham joints had a smooth surface and featured no signs of OA (left). Slides of the top three rows were stained with Safranin O/Fast Green. In the bottom row, further examples of the cartilage surface after staining with hematoxylin and eosin are shown. **c** Immunohistochemistry with an antiserum raised against the C1,2C collagen cleavage neoepitope was performed to confirm matrix metalloproteinase (MMP)-mediated extracellular matrix degradation and thus development of an OA-like phenotype in response to the DMM treatment. Here, positive staining at the articular cartilage surface could be detected in the DMM joints (arrowheads), whereas this was not the case in the sham joints. The specificity of the staining was controlled with *Tfap2e* ^−/−^ sections without primary antibody treatment (indicated by a “-”), and sections of human OA cartilage were used as positive controls. **d** Total RNA from the murine articular cartilage layer was isolated 10 and 17 days after OA induction, and expression of activating enhancer binding protein 2, epsilon (AP-2ε) was analyzed in knee joints of WT mice 10 and 17 days after OA onset relative to untreated WT knee joints. A significant induction in the expression rate of the transcription factor could be measured in the articular cartilage of the DMM joints. The data are given as box-and-whisker plots with minimum and maximum values in (a) and as the means ± standard errors of the mean in (d). ns, Not significant; *Tfap2e* ^−/−^, Deficient for activating enhancer binding protein 2, epsilon. *P < 0.05; **P < 0.01; ***P < 0.001. Numbers indicate individual animals per group

OA was induced in the right hind limbs of 6- to 8-week-old animals by DMM from the tibial plateau; the left hind limbs served as controls (sham). The progression of OA was analyzed 10 and 17 days after surgery. As depicted in Fig. 6a, animals of both genotypes exhibited only minor signs of OA 10 days after disease onset. Seventeen days after surgery, the WT mice showed mild to moderate signs of OA, as expected on the basis of previous studies [27] (Fig. 6a). These included wear, lesions and inhomogeneous staining near the cartilage surface (Fig. 6b). In striking contrast, both the severity and the extent of the cartilaginous destruction were significantly enhanced in *Tfap2e*^−/−^ mice at the corresponding juncture (Fig. 6a and b). In all of the sham joints, the articular cartilage layer had a smooth surface contour and manifested no signs of OA (Fig. 6b). To substantiate that the DMM-treated joints exhibit an OA phenotype and to confirm involvement of MMPs in this process, immunostaining for collagenase-induced collagen breakdown products was performed using an antiserum against the C1,2C cleavage neoepitope. A clear positive staining signal was detectable at the surface of the articular cartilage layer in the DMM joints of both WT and *Tfap2e*^−/−^ animals (Fig. 6c). In contrast, no staining could be determined in the cartilage surface of the sham joints. The specificity of the staining was controlled with murine sections without primary antibody treatment used as negative controls and sections of human OA cartilage used as positive controls.

**Fig. 7:**
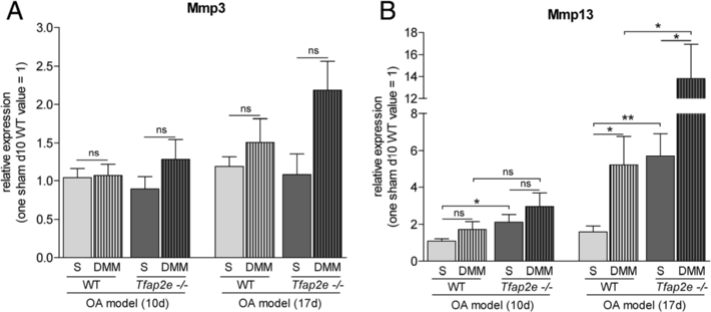
Analysis of matrix metalloproteinase expression during osteoarthritis progression. Total RNA from the articular cartilage layer was isolated 10 and 17 days after osteoarthritis (OA) induction by detaching the medial meniscus (DMM), and expression of the matrix metalloproteinase 3 (Mmp3) **(a)** and Mmp13 **(b)** was analyzed by quantitative RT-PCR. (A) Mmp3 mRNA expression was approximately identical in all sham joints (S) at both time points, and only a minor induction could be detected in the DMM joints compared with the sham joints. (b) In contrast, a significant higher basal level of Mmp13 expression could be detected in the sham joints of the *Tfap2e* ^−/−^ mice compared with the sham joints of the wild-type (WT) mice at both time points. In addition, the expression of Mmp13 was significantly upregulated in the DMM joints compared with the sham joints 17 days after OA induction in both genotypes. In combination with the higher basal expression rate, this resulted in a significantly increased total Mmp13 expression level in articular chondrocytes of the *Tfap2e* ^−/−^ mice at day 17 after OA onset. The data are given as the means ± standard error of the mean. ns, Not significant; *Tfap2e* ^−/−^, Deficient for activating enhancer binding protein 2, epsilon. *P < 0.05; **P < 0.01. Five to eight animals were used for each group

In addition to histology, we isolated RNA from the articular cartilage of the knee joints of the mice in the OA model. As detected in patients with OA compared with healthy donors [21], a significant upregulation of *AP-2ε* expression was measured in the joints subjected to OA surgery compared with the untreated joints in WT mice (Fig. 6d). *Mmp1* was not detectable in murine articular chondrocytes even after OA onset (data not shown). For *Mmp3*, we also did not observe differential expression in *Tfap2e*^−/−^ mice compared with WT mice. Furthermore, no significant changes in *Mmp3* expression in the DMM joints compared with the controls could be determined at either time point (Fig. 7a). For *Mmp13*, again, a significantly higher basal expression level in AP-2ε-deficient articular chondrocytes could be detected when we compared the sham joints of *Tfap2e*^−/−^ mice with the sham joints of WT mice at both time points (Fig. 7b). In addition, in both genotypes, the expression of *Mmp13* was significantly upregulated in articular cartilage derived from the DMM joints compared with the sham controls 17 days after disease onset. This was expected owing to the pathological conditions [13–15]. However, in combination with the higher basal Mmp13 expression rate, this resulted in a significantly increased total *Mmp13* expression level at day 17 after OA onset in the *Tfap2e*^−/−^ mice. This finding most likely explains the enhanced OA manifestation observed in these animals and shows that AP-2ε is involved in progression of the disease.

## Discussion

Recently, we discovered physiological expression of the transcription factor *AP-2ε* in hypertrophic chondrocytes as well as in adult articular cartilage [20, 21]. Adult mice deficient for *AP-2ε*, however, do not exhibit an apparent cartilaginous or skeletal phenotype compared with WT mice. Nevertheless, as observed in other murine knockout models of molecules important for cartilage development, it could be possible that abnormalities arise only in the embryo during chondrogenesis and are compensated later in development via redundancy or irregular cellular differentiation and proliferation. For instance, mice with a cartilage-specific knockout of *Mmp13* show a significantly increased width of the hypertrophic zone in the growth plate during embryonic and early postnatal development [35]. This phenotype completely resolves at approximately 12 weeks of age, and adult *Mmp13*^−/−^ mice display no phenotypic abnormalities [35]. Another example for redundancy during chondrogenesis is the mouse deficient for melanoma inhibitory activity/cartilage-derived, retinoic acid–sensitive protein (MIA/CD-RAP), which also does not manifest strong abnormalities [31]. Nevertheless, recent studies by our group revealed enhanced proliferation and delayed differentiation in *MIA*^−/−^ mouse embryos at E15.5/E16.5, resulting in abnormal growth plate architecture. This is compensated shortly afterward, however, possibly via reduced cAMP response element-binding protein and activator protein 1 (AP-1) activity [36].

Thus, in the first part of this study, we compared the embryonic skeletal development of *Tfap2e*^−/−^ and WT mice to find transient differences in chondrogenesis and endochondral ossification, respectively. Those differences, in turn, could provide valuable information about the influence of the transcription factor in these processes. Advanced stages of skeletal development (E15.5/E16.5) were assessed *in vivo* via measurement of the embryos’ weight, size and femoral lengths, as well as by determination of the femoral expression zones of *Col2a1* and *Col10a1*. In addition, early chondrogenesis was monitored *in vitro* via micromass cultivation of mesenchymal cells that were isolated from limb buds of E11.5 WT and *Tfap2e*^−/−^ mouse embryos. Taken together, no fundamental abnormalities in the embryonic development of *Tfap2e*^−/−^ mice became apparent in these experiments. However, at E16.5, a small, albeit significant, increase in the *Col10a1* expression zone, which is specific for highly differentiated hypertrophic chondrocytes [37–39], was observed. Similarly, the *Col10a1* transcription rate was upregulated in AP-2ε-deficient limb bud cells at very late stages of micromass differentiation. Likewise, the expression of *Mmp13* tended to be enhanced in those cells, which suggests that loss of AP-2ε results in enhanced expression of these hypertrophic markers [37, 39, 40]. Again, the observed discrepancies were of rather low magnitude, and no differences could be observed earlier during chondrogenesis, although the mRNA expression of *AP-2ε* was clearly detectable in the mesenchymal cells. One possible explanation for these findings is that the loss of *AP-2ε* is compensated by an enhanced activity of redundant factors. Here, other *AP-2* isoforms expressed during chondrogenesis come into consideration. For instance, Huang *et al.* provided evidence that *AP-2α* is a negative regulator of chondrocyte differentiation [41]. Likewise, *AP-2β* expression was detected in the developing mouse limb [42]. However, when *AP-2ε* is highly expressed in differentiated chondrocytes, the compensatory mechanisms may not be sufficient to fully negate the loss of the transcription factor. Alternatively, it could also be possible that *AP-2ε* does not impact gene expression in mesenchymal cells and early chondroblasts, because of either a lack of additional factors that are essential for AP-2ε function or the general low level of expression of AP-2ε in these cells. Regarding the latter, we determined a strong induction of AP-2ε expression in articular cartilage tissue isolated from adult mice compared with mesenchymal limb buds in WT animals.

We therefore concentrated on AP-2ε in adult joint cartilage. The focus was on *Col10a1* and *Mmp13*, as these genes already showed differential expression during late stages of *in vitro* differentiation of mesenchymal cells of WT and *Tfap2e*^−/−^ mice. Indeed, we found Mmp13 expression to be significantly upregulated in articular cartilage isolated form *Tfap2e*^−/−^ mice compared with WT animals, whereas Col10a1 was not deregulated. Although we detected *Col10a1* mRNA, the latter is most likely attributable to the fact that expression of *Col10a1* in articular cartilage is restricted to single cells in the calcified deep zone and thus is generally very low in this tissue type [38, 43].

Mmp13 is known to be constitutively expressed in articular cartilage at low levels during physiologic ECM turnover [8, 14, 16, 17] and is capable of degrading native collagen type II [14, 44, 45] and aggrecan [46–48]. In addition, the family of matrix metalloproteinases comprises crucial mediators of cartilage destruction in OA. Of those, MMP13 is the most important, being overexpressed in the affected tissue of most patients with OA. Other variants, such as MMP1 and MMP3, also were shown to play a role in some cases of human OA [7, 13, 14, 19]. Furthermore, Mmp activity is strongly modulated at the posttranscriptional level via catalytic processing and Timp1, Timp 2 and Timp 3 [45]. Expression of the latter was unchanged in *Tfap2e*^−/−^ mice, revealing that Timps do not counterbalance the enhanced *Mmp13* mRNA expression [49, 50].

Furthermore, Mmp13 activity was directly measured in supernatants of articular cartilage explants and was indeed significantly enhanced in *Tfap2e*^−/−^ mice. To determine the functional correlation, GAG release was assessed, which we found to be significantly higher in media from AP-2ε-deficient explants. A specific Mmp13 inhibitor confirmed that Mmp13 was mainly responsible for these observations because, after addition of the compound, the prior significant difference between the two genotypes was abolished.

Unexpectedly, *in vivo* the articular cartilage tissue of untreated 10.5-week-old *Tfap2e*^−/−^ mice exhibited no evident abnormalities. In striking contrast, profound differences compared with WT mice became obvious after OA induction via DMM surgery. In one of our previous studies, the same OA model was carried out with MIA/CD-RAP deficient mice of a similar age. Here, slight signs of OA were detectable 10 days after surgery, and moderate signs were detectable 21 days after surgery [27]. Accordingly, comparable OA development could be observed in this study after 10 and 17 days. Again, in this model, a significantly higher basal level of *Mmp13* expression in AP-2ε-deficient articular chondrocytes could be confirmed when we compared the sham joints of both genotypes. This, in combination with the characteristic OA-dependent induction of *Mmp13* expression, resulted in a significantly increased total *Mmp13* expression level in the *Tfap2e*^−/−^ mice at day 17 after disease onset, which most likely is responsible for enhanced OA severity in these animals. Other examples of genetically manipulated mice that were deficient for cartilage-associated molecules and did not exhibit phenotypic alterations in the adult stage, but nevertheless responded to pathologic conditions or tissue stress differently from the WT mice, including mice deficient for Adamts5 (a disintegrin and metalloproteinase with thrombospondin motifs 5), MIA/CD-RAP and Mmp13 [27, 32–34]. In indirect correlation with our results, OA severity was significantly reduced in the latter mice. Further analyses in *Mmp13* transgenic mice revealed that a constant overexpression of Mmp13 in articular chondrocytes could indeed promote disease progression [51], thus confirming that a dysregulation of *Mmp13*, as observed in the *Tfap2e*^−/−^ mouse, strongly influences OA development.

**Fig. 8:**
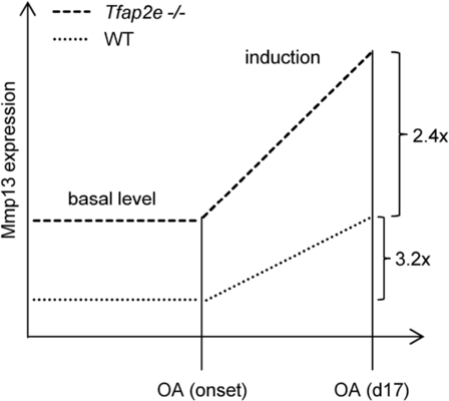
Graphical illustration of the results. Our data suggest that the loss of activating enhancer binding protein 2 epsilon (AP-2ε) results in an enhanced basal matrix metalloproteinase 13 (Mmp13) expression level in articular chondrocytes of *Tfap2e* ^−/−^ mice as compared with wild-type (WT) mice. After osteoarthritis (OA) onset, expression of Mmp13 is induced in both genotypes by a comparable amount (3.2-fold in WT mice and 2.4-fold in *Tfap2e* ^−/−^ mice), which is most likely due to the effect of other OA-associated transcription factors. However, because of the higher basal expression level, a similar induction rate leads to a significantly higher Mmp13 expression level in chondrocytes of *Tfap2e* ^−/−^ mice compared with WT mice 17 days after OA surgery. *Tfap2e* ^−/−^, Deficient for activating enhancer binding protein 2, epsilon

In summary, the obtained data suggest that AP-2ε effectively represses basal *Mmp13* expression under normal, non-OA conditions in articular chondrocytes of WT mice and that this control mechanism is lost in *Tfap2e*^−/−^ animals. However, despite this inhibitory effect, an induction of *Mmp13* expression after OA onset could be measured not only in *Tfap2e*^−/−^ mice but also in WT animals. It is known that changes in the gene expression profile of the affected chondrocytes take place during OA, including upregulation of factors that drive expression of various effector molecules such as Mmps. For example, *Mmp13* expression was shown to be upregulated by Runx2 (runt-related transcription factor 2), AP-1, HIF-2α (hypoxia-inducible factor 2, alpha) and Ets (E26 transformation-specific) family members [52–56]. As *Mmp13* expression is similarly induced in both genotypes after OA onset (about 3.2-fold in WT mice and 2.4-fold in *Tfap2e*^−/−^ mice), the inhibitory effect of AP-2ε on *Mmp13* in the WT animals must be overbalanced by one or more of these activating factors. However, owing to the significantly higher basal expression, the induction results in a significantly higher *Mmp13* expression level in articular chondrocytes of *Tfap2e*^−/−^ mice compared with WT mice also at day 17 after OA surgery (Fig. 8). As demonstrated in the OA model, this increases the susceptibility of the cartilage tissue to destruction after joint overload, resulting in enhanced OA severity in the *AP-2ε*-deficient animals.

As determined *in vitro*, the higher basal expression and activity of Mmp13 in articular chondrocytes of *Tfap2e*^−/−^ mice shifts physiologic matrix turnover to the catabolic side also under non-OA conditions. Hence, it is possible that *Tfap2e*^−/−^ mice develop OA-like symptoms with advanced age. In *Mmp13* transgenic mice that strongly overexpress Mmp13 in articular chondrocytes, cartilage destruction was apparent at 5 months of age. However, it could also be possible that the loss of *AP-2ε* is compensated in the *Tfap2e*^−/−^ animals via unknown mechanisms later during development. For this study, all experiments were carried out with mice up to 12 weeks of age, and further investigations with older mice of 6 to 12 months of age will be very interesting.

The expression of AP-2ε was induced in joints subjected to OA surgery compared with untreated joints in WT mice, which was similarly observed in humans [21]. This induction may constitute an attempt of chondrocytes to prevent the activation of *Mmp13* after initial OA onset. However, at the same time, other regulatory factors drive the expression of *Mmp13* and eventually predominate over AP-2ε. Thus, the increase of AP-2ε expression might only be sufficient to delay OA progression, but not to fully prevent cartilage destruction, which is in line with our observations in the OA model.

## Conclusions

Despite the publication of large numbers of scientific reports, numerous aspects of cartilage development, destruction and regeneration are still not fully understood. Hence, studies introducing new players or interactions influencing these complex processes are of great importance. In this work, we reveal a novel role of the transcription factor AP-2ε in the control of articular chondrocyte gene expression and OA development by influencing *Mmp13* expression and activity *in vivo*. Thus, this finding adds to the understanding of regulatory mechanisms underlying OA and might influence the development of future treatment options.

## Abbreviations

AC: Articular chondrocytes
AP-1: Activator protein 1
AP-2: Activating enhancer binding protein 2
*Col2a1*: Collagen, type II, alpha 1
*Col10a1*: Collagen, type X, alpha 1
CXCL1: Chemokine (C-X-C) motif ligand 1
DMEM: Dulbecco’s modified Eagle’s medium
DMM: Detaching the medial meniscus
E: Embryonic day
ECM: Extracellular matrix
FCS: Fetal calf serum
GAG: Glycosaminoglycan
LB: Limb bud cells
MIA/CD-RAP: Melanoma inhibitory activity/cartilage-derived, retinoic acid–sensitive protein
Mmp: Matrix metalloproteinase
OA: Osteoarthritis
PBS: Phosphate-buffered saline
qRT-PCR: Quantitative real-time polymerase chain reaction
*Tfap2e*: Transcription factor activating enhancer binding protein 2 epsilon gene
Timp: Tissue inhibitor of metalloproteinase
WT: Wild type
